# Understanding Behavioral Intentions Toward COVID-19 Vaccines: Theory-Based Content Analysis of Tweets

**DOI:** 10.2196/28118

**Published:** 2021-05-12

**Authors:** Siru Liu, Jialin Liu

**Affiliations:** 1 Department of Biomedical Informatics University of Utah Salt Lake City, UT United States; 2 Department of Medical Informatics West China Hospital Sichuan University Chengdu China

**Keywords:** vaccine, COVID-19, behavior, tweet, intention, content analysis, Twitter, social media, acceptance, threshold, willing, theory, model, infodemiology, infoveillance

## Abstract

**Background:**

Acceptance rates of COVID-19 vaccines have still not reached the required threshold to achieve herd immunity. Understanding why some people are willing to be vaccinated and others are not is a critical step to develop efficient implementation strategies to promote COVID-19 vaccines.

**Objective:**

We conducted a theory-based content analysis based on the capability, opportunity, motivation–behavior (COM-B) model to characterize the factors influencing behavioral intentions toward COVID-19 vaccines mentioned on the Twitter platform.

**Methods:**

We collected tweets posted in English from November 1-22, 2020, using a combination of relevant keywords and hashtags. After excluding retweets, we randomly selected 5000 tweets for manual coding and content analysis. We performed a content analysis informed by the adapted COM-B model.

**Results:**

Of the 5000 COVID-19 vaccine–related tweets that were coded, 4796 (95.9%) were posted by unique users. A total of 97 tweets carried positive behavioral intent, while 182 tweets contained negative behavioral intent. Of these, 28 tweets were mapped to capability factors, 155 tweets were related to motivation, 23 tweets were related to opportunities, and 74 tweets did not contain any useful information about the reasons for their behavioral intentions (κ=0.73). Some tweets mentioned two or more constructs at the same time. Tweets that were mapped to capability (*P*<.001), motivation (*P*<.001), and opportunity (*P*=.03) factors were more likely to indicate negative behavioral intentions.

**Conclusions:**

Most behavioral intentions regarding COVID-19 vaccines were related to the motivation construct. The themes identified in this study could be used to inform theory-based and evidence-based interventions to improve acceptance of COVID-19 vaccines.

## Introduction

The global COVID-19 pandemic is affecting 219 countries worldwide [1]. An important component of managing COVID-19 is preventing the infection [2,3]. Fortunately, development of vaccines against the disease is progressing well. In December 2020, the US Food and Drug Administration authorized COVID-19 vaccines for emergency use. Another currently pressing issue is how to increase vaccine acceptance rates [4]. In previously published survey studies, the acceptance rate of COVID-19 vaccines was a concern. Of 672 participants in the United States, approximately 67% said they would be willing to receive a vaccine [5]. It is necessary to vaccinate an estimated 55%-82% of the population to create herd immunity and slow the spread of a pandemic [6]. Therefore, it is critical to understand why some people are willing to be vaccinated and others are not.

Previous studies have described potential impediments to COVID-19 vaccines, including questioning the need for vaccines and preferring to benefit from the immunity conferred by surviving COVID-19 [7]; safety issues regarding the rapid development and testing process of vaccines [7]; issues related to mandatory vaccination [7]; and conspiracy beliefs [8]. Some researchers conducted surveys based on theoretical models to explore facilitators and barriers to COVID-19 vaccination. Williams et al [9] conducted a survey to examine factors that influenced respondents’ decisions to be vaccinated against COVID-19 and identified three facilitators (personal health, severity of COVID-19 disease, health consequences to others) and one barrier (concerns regarding vaccine safety). Lin et al [10] used the health belief model (HBM) to identify two facilitators (reduced likelihood of contracting COVID-10; others getting vaccinated) and one barrier (concerns about efficacy and side effects). Wong et al [11] also used the HBM to identify one facilitator of perceived benefits (the belief that the vaccination can reduce infection probability and alleviate concerns about COVID-19).

Compared with surveys, Twitter can gather timely information regarding behavioral intentions toward COVID-19 vaccines, especially to understand “anti-vaxxers” and those influenced by misinformation who are inclined not to get the vaccine. These types of users could be the most vulnerable population for COVID-19 vaccine outreach interventions. On the Twitter platform, there are more than 330 million users, and the median number of posts per person each month is 2 [12,13]. A recent survey of US Twitter users showed that they are younger and have a higher education level than the general population; however, their gender, race, and ethnicity distributions are similar to those of the general population [13]. The Twitter platform has been validated as a way to develop a public perception–tracking tool based on real-time content [14]. In addition, Twitter has been flooded with information about COVID-19, influenced by social isolation policies enacted during the epidemic [15]. The Twitter platform could be used to explore determinants of health-related behavior intentions [16]. Because the maximum length of each tweet is 280 characters, in addition to mentioning potential behavioral intentions, users can also briefly describe the reasons that led to their decision. In addition, using geotagged Twitter data, it is easier and faster to identify people's perceptions in different geographic locations. Therefore, we chose to use the Twitter platform to analyze behavioral intentions toward COVID-19 vaccines.

**Figure 1:**
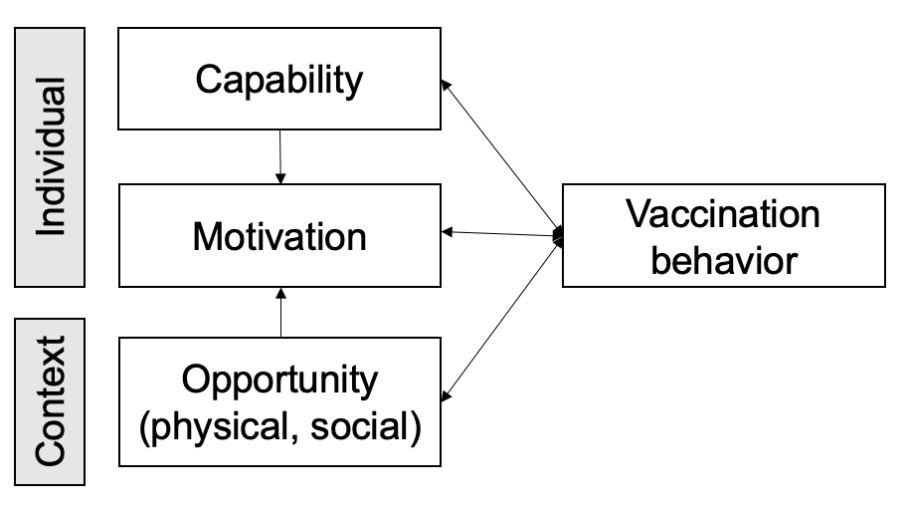
The theoretical model based on the capability, opportunity, motivation—behavior (COM-B) model adapted to vaccination behavior, developed by the World Health Organization Regional Office for Europe [24,25].

To better characterize the factors that influence behavioral intentions on the COVID-19 vaccines mentioned in the tweets, we conducted a theory-based content analysis based on the capability, opportunity, motivation–behavior (COM-B) model. The COM-B model was proposed by Michie et al [17] in 2011, and it contains three basic constructs: capability (physical and psychological), motivation (automatic and reflective), and opportunity (social and physical). The COM-B model is a comprehensive theoretical model based on causal mechanisms to identify individual and context factors that influence behavioral change. It was developed by merging 19 behavior change frameworks through a systematic literature review and discussions with behavior change experts. It has been successfully applied to many health-related behaviors, such as smoking cessation [18-20] and obesity reduction [21,22]. In contrast to other health behavior theories (eg, HBM, theory of planned behavior), the COM-B model was developed based on the behavior change wheel, which not only provides a theoretical analysis of behavior but, more importantly, provides results that can be used to assist with intervention design [17,23]. In addition, the World Health Organization (WHO) Regional Office for Europe has adapted it to vaccination behaviors to design its Tailoring Immunization Programmes (TIP) approach [24,25]. They merged subconstructs in capability and motivation, respectively. Because of vaccination behavior, the physical capability is interlinked with the psychological capability. Likewise, automatic motivation (ie, emotions, impulses) interacts with reflective motivation (ie, intentions, beliefs). Another advantage of the adapted COM-B model is that it focuses on vaccination behavior and provides refined details for each construct in the vaccine context. Therefore, we used an adapted COM-B model (see Figure 1) as the theoretical model of this study. The objectives of this study are to (1) determine if the adapted COM-B model can explain behavioral intentions toward COVID-19 vaccines using tweets; (2) examine theory-informed factors that may affect behavioral intentions toward COVID-19 vaccines; and (3) extract themes to provide information for public health researchers to develop theory-based and evidence-based promotion interventions.

## Methods

### Adapted COM-B Model

The adapted COM-B model has three theoretical constructs: (1) capability, (2) motivation, and (3) opportunity. The WHO Regional Office for Europe also provided examples for each construct (see Table 1). Capability refers to the individual’s ﻿physical and psychological ability to perform the behavior, along with the knowledge and skills required to complete the activity [17]. In particular, psychological ability is the ability of an individual to have the necessary thought processes, such as being able to understand, reason, etc [17]. Motivation has a broad definition that includes goals and conscious decision-making as well as all other individual processes that motivate and lead to behavior, such as automatic processes (habitual processes, emotional responses) and reflective processes (analytical decision-making) [17]. Opportunity refers to the contextual factors that prompt the behavior to occur, including the physical opportunity and the environment and social opportunity [17]. The adapted COM-B model also provides a dynamic relationship between constructs. For example, both capability and opportunity can affect motivation. All three constructs of competence, motivation, and opportunity can generate behavior; on the other hand, behavior can influence these three constructs.

**Table 1:** The theoretical constructs in the adapted capability, opportunity, motivation–behavior (COM-B) model and associated examples by the World Health Organization Regional Office for Europe.

Theoretical construct	Examples
Capability	Knowledge Skills, trust in own skills Resilience, stamina, will power, surplus energy Physical fitness, ability
Motivation	Attitudes, perceptions, risk assessment Values, beliefs Emotions, impulses, feelings Confidence, trust
Opportunity (physical)	Access, affordability, availability of vaccination Convenience, appeal, appropriateness of vaccination Structural efficiency Availability of information
Opportunity (social)	Social, cultural demands, support Social, cultural cues, norms, values

### Data Collection

We collected English tweets posted from November 1-22, 2020, using a combination of relevant keywords and hashtags: (#covid OR covid OR #covid19 OR covid19) AND (#vaccine OR vaccine OR #vacine OR vacine OR vaccinate OR immunization OR immune OR vax). After excluding retweets, we randomly selected 5000 tweets for manual coding and content analysis. The random numbers were generated through the NumPy package in Python. Then, we mapped the random numbers with the index of collected tweets.

### Content Analysis

We performed a content analysis informed by the adapted COM-B model. The coding schema was developed iteratively. First, we developed the coding schema based on the definitions of constructs in the adapted COM-B model. Two reviewers (SL and JL) independently coded 1000 tweets in each round. After completing one round of coding, the two reviewers met with a third reviewer to discuss disagreements and update the coding schema until consensus was reached. We calculated the interrater reliability for the last round. If a tweet mentioned ≥2 constructs simultaneously, we coded it with multiple labels. We conducted chi-square tests to explore the relationship between theoretical constructs with the positive/negative behavioral intention. The statistical significance threshold was .05.

## Results

### Data Collection

**Figure 2:**
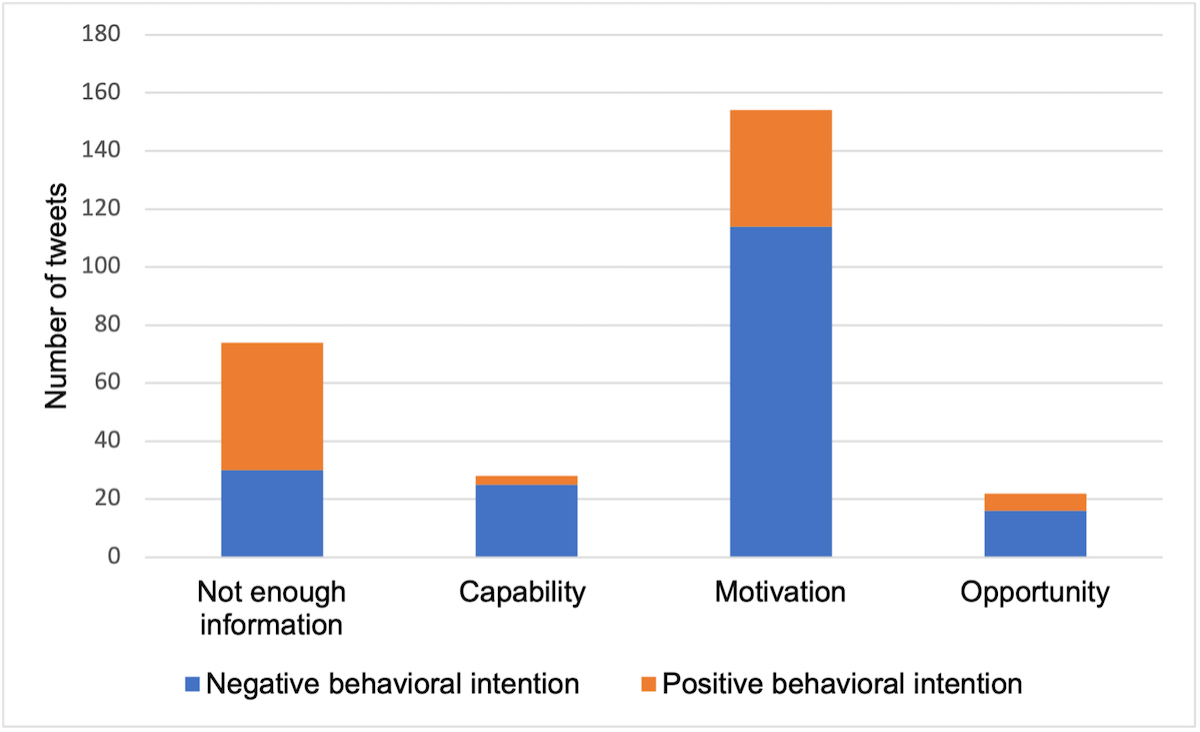
Numbers of tweets containing different theoretical constructs.

We coded 5000 COVID-19 vaccine–related tweets, which were posted by 4796 unique users. We found 279 tweets that stated their behavioral intentions. The remaining tweets did not state any behavioral intentions toward COVID-19 vaccines. A total of 97 tweets were labeled with positive behavioral intentions, while 182 tweets contained negative behavioral intentions. Among them, 28 tweets were mapped with capability factors; 155 tweets were related to motivation; 23 tweets were related to opportunities; and 74 tweets did not contain any useful information about reasons for their behavioral intentions (see Figure 2). The κ value was 0.73. Of the tweets, 2 mentioned ≥2 constructs at the same time. Tweets that mentioned capability (χ^2^_1_=17.286, *P*<.001), motivation (χ^2^_1_=35.558, *P*<.001), and opportunity (χ^2^_1_=4.545, *P*=.03) were more likely to have negative behavioral intentions (Table 2).

**Table 2:** Themes and example tweets (n=204).

Theme			Value, n (%)		Example tweets
**Capability (n=28)**					
	Knowledge	24 (11.8)		“I am not getting a covid vaccine because there will be a microchip in there so the government can track my weekly shop in Lidls” “No way I'm taking it. In one study out of women became steril [sic] from taking the vaccine. Is this the one that alters your DNA and can cause cancer? Thanks but I would rather get COVID.” “As I said it's not a 'from scratch' situation...covid is one version of a virus that a lot of work has already been done on. It's just a case of isolating the correct strain to vaccinate against. Look if you don't want to get it fine...all the more for me and my family.”	
	Physical condition	4 (2)		“I got my flu shot a few days ago. For now there is no way I will get a COVID vaccine.” “I have severe adverse reactions to vaccines and I meet of the exclusion criteria for the COVID vaccine trials.”	
**Opportunity (n=22)**					
	Rights, regulation and legislation (eg, charging for vaccination, mandatory vaccination, inadequate regulation of vaccines, poor availability of information)	11 (5.4)		“Tbh^a^ I'm not against every vaccine in history. Just this specific COVID-19 vaccine. There are many reasons why, for example the nonsensical social regulations, that people like you probably want to be over so bad. But you seem to hot headed to absorb any legit information.” “If they charge us for this vaccine. I don't give. Not a single person should have to pay for a Covid Vaccine.” “Please stop volunteering me for this COVID-19 vaccine. I need more information. I've been close enough to death this year, no thank you. -a healthcare worker.”	
	Social and cultural demands and support	9 (4.4)		“Someone that works for the CDC^b^ told me, do NOT get the covid vaccine.. Not sure but I'm not OK with her saying that at all.” “I'm not getting a covid vaccine. Do what yall want with that info to write me off. But its against my culture and everything I've been practicing.”	
	Social consequences and reactions to vaccination	2 (1)		“when anti vaccs realize everything is gonna start requiring the covid vaccine i can't wait to see. and for natural selection to do what it does best (unless you're immunocompromised and can't take certain vaccines, then they'll accept testing of course)”	
**Motivation (n=154)**					
	Attitudes and perceptions about the COVID-19 vaccine or the disease itself (eg, disease severity, vaccine effectiveness)	68 (33.3)		“Yeah, with an average age of over you forgot to mention, a minute percentage of those that have had it and almost elderly and/or with underlying health conditions. For many of us, taking the rushed vaccine may more likely to cause us harm than covid itself” “OK. so we each make our own risk/benefit decision. As the virus is what, % fatality, why would someone choose an unproven mRNA vaccine, with phase trials only since July? a biotech never before used programming RNA transcriptase to produce Covid in your cells? No thx.”	
	Strong emotions about COVID-19 vaccine (eg, fear of side effects, unfairness to COVID-19 survivors)	5 (2.4)		“We shouldn't have a COVID vaccine because it'll make those who lost loved ones to COVID angry.”	
	Values and beliefs (eg, natural immune system, alternative medicine, value of vaccination)	37 (18)		“My immune system is better than any vaccine. And a vaccine works in tandem with the way how your immune system beats virus No COVID Vax for me!” “I had covid19 because I'm a long haul trucker and travel all over to different states..what I did to cure myself was take % alcohol and pour it on a rag and breathing in the fumes before I went to bed..I did that minutes a night..I got well in no time.. don't need no vaccine”	
	Confidence and trust (vaccine, health authorities, science, and government)	13 (6.3)		“I do not trust the FDA^c^, especially concerning the brand new Covid vaccine being rushed to market. Covid poses almost zero risk to my demographic. The pharmaceutical companies face zero risk if the vaccine damages their customers. For me the vaccine is riskier than the virus.” “I won't accept COVID vaccine offered by Biden administration...”	

^a^Tbh: to be honest.

^b^CDC: US Centers for Disease Control and Prevention.

^c^FDA: US Food and Drug Administration.

### Capability

The first theme regarding capability was that some users lacked knowledge about the COVID-19 vaccines and were influenced by misinformation. mRNA vaccines are a new technology, and some believe that these vaccines can alter DNA. As for the vaccine itself, some users believe that it contains a microchip that can be tracked by the government. Some people expressed that they did not know the side effects of COVID-19 vaccines or even their long-term effects. Based on this concern, misinformation was generated that the vaccine could cause sterility, cancer, etc. Other reasons for not getting the vaccine included the belief that the vaccine could make people sick, the belief that there is no need for people who have been infected with COVID-19 to get the vaccine, and that the vaccine does not prevent infection but only alleviates symptoms. All these beliefs reflect a lack of understanding of the COVID-19 vaccines among users. Having more knowledge about vaccines could help people develop positive behavioral intentions; for example, some people mentioned that they could understand why the development process was fast because researchers only needed to isolate the correct strain rather than create one. Others mentioned understanding that vaccines do not contain live viruses, so they would be willing to get the vaccine.

Some users emphasized that their physical condition was not suitable for COVID-19 vaccination and that they were unsure how their body would react after vaccination (eg, recently had an influenza vaccine shot, had a suppressed immune system, had a severe adverse reaction to vaccination in the past, had a stroke).

### Opportunity

In the physical opportunities category, some users said they would not accept the vaccine if they had to pay for it. Many users said vaccination should be a free choice and that they would refuse to receive the vaccine if it became mandatory. Some users wanted more regulation of the COVID-19 vaccine, and some were concerned about the availability of information.

Among the social opportunities, we found some factors influencing the COVID-19 vaccination related to social and cultural demands and support, such as going against religion, defying culture, health workers not recommending vaccination, and family members actively discouraging vaccination. Others intended to be vaccinated because of the fear of the social consequences and reactions to vaccination, such as fear of affecting their work and requiring proof of vaccination for many activities in the future.

### Motivation

Most of the reasons for behavioral intentions were categorized into the motivation construct. Many users expressed attitudes and opinions about the COVID-19 vaccine or the disease itself, such as not considering the disease to be severe or life-threatening and not considering the vaccine to be effective (because of low efficiency and mutation of the virus). Others assessed the risks and considered the rushed vaccine to be more harmful than COVID-19. Some of those with positive behavioral intentions stated that they chose vaccination because they did not want to be infected with COVID-19.

Some users expressed strong emotions and feelings about COVID-19 vaccines, such as fear of the vaccine and concern about its side effects because it is new. Notably, others felt that vaccination was selfish or unfair, as they would not be exposing themselves to the same risk as others who survived COVID-19; thus, they were reluctant to be vaccinated.

﻿Other themes were values and beliefs. Some users expressed a belief that the body’s natural immune system is better than any vaccine, or they believed more in alternative medicine. On the other hand, others who wanted to be vaccinated emphasized the positive values of vaccination, such as saving more lives, reopening the economy, and returning to normal life.

﻿Confidence and trust were dominant themes. Users expressed distrust in many areas: the quality of the vaccines (hastily manufactured, untested), health authorities, science, companies, and government. For Twitter users in the United States, we found that unlike with other vaccines, part of the reason people do not trust the government is because of their past handling of the COVID-19 pandemic. In addition, the COVID-19 vaccine rollout came during a time when a new president was being elected in the United States, and some people lacked confidence in the newly elected president’s party.

## Discussion

### Principal Findings

In this study, we conducted a theory-based content analysis using a dataset of 5000 tweets posted from November 1-22, 2020. We identified 279 tweets that contained behavioral intentions regarding COVID-19 vaccines and mapped them to constructs in the adapted COM-B model. We generated nine themes that influence Twitter users’ intentions to receive COVID-19 vaccines. The constructs in the COM-B model could be applied systematically to characterize factors that influence behavioral intentions toward COVID-19 vaccines. In addition, we found that among tweets that simply stated behavioral intentions without including any reason, the number of positive intention tweets was higher than that of negative intention tweets. The results also implied that more than half of the tweets expressing the decision-making process were negative intention tweets. This finding aligns with our expectation of understanding the factors that contribute to vaccine hesitancy to better develop tailored vaccine promotion programs.

The novelty of COVID-19 vaccines and the current social context have created further difficulties in vaccine rollout. Identified barriers of influenza vaccination intention and behavior include lack of confidence (eg, negative attitudes, mistrust), inconvenience (eg, cost, access), calculation (eg, risk assessment), and complacency (eg, underestimating disease severity) [26]. Our results revealed the presence of several other factors that influence behavior toward COVID-19 vaccination. First, misinformation or conspiracy theories about COVID-19 and COVID-19 vaccines are much more prevalent on social media than those about other diseases or their related vaccines [27-29]. Some users were influenced by this misinformation, and this led to refusal of the vaccine. Second, Twitter users expressed concerns about mandatory vaccination. At the Emergency Use Authorization stage, mandatory vaccination is legally and ethically questionable [30]. However, with full Biologics License Application approval, policy makers may mandate vaccination for all populations. Given the existence of users who have strongly indicated that they would not accept mandatory vaccination against COVID-19, policy makers must be cautious in determining vaccination policies for the public. Some studies have suggested that vaccine mandates do not improve vaccine acceptance rates, and a proposed alternative approach is to apply informed risk communication and give people the freedom to choose without compromising personal autonomy [30]. Third, users with positive behavioral intentions emphasized that the positive value of vaccination to society, such as restoring economic and normal life from before the epidemic, motivated them to be vaccinated. This facilitator is uncommon in other vaccination behaviors. This facilitator could be matched with a strategy of converting personal decisions into public acts [31]. Fourth, we observed that some users were reluctant to be vaccinated because they felt that the COVID-19 vaccine was unfair to those who survived. Based on this concern, it may be helpful to select COVID-19 survivors as opinion leaders to promote COVID-19 vaccines, increasing public awareness of the severity and risk of the disease. Fifth, in addition to the mistrust of vaccines and science that also exists for other vaccines, for the COVID-19 vaccine, users in the United States expressed more mistrust of the government for two specific reasons: (1) the previous administration's inappropriate behavior in handling COVID-19 and (2) the lack of confidence in the newly inaugurated president’s political party. These findings were also aligned with previous studies that proposed a role of politics in COVID-19 vaccine hesitancy [8]. Sixth, it is worth noting that even though previous studies have identified that past vaccination behavior can be used to predict future vaccination behavior [32], we found that past experiences with other vaccines may not affect COVID-19 vaccination. For example, one user mentioned:

I'm not against every vaccine in history. Just this specific COVID-19 vaccine. There are many reasons why, for example the nonsensical social regulations.

The above differences contribute to the fact that the rollout of COVID-19 vaccines could be more complicated than that of other vaccines. Researchers need to develop interventions specific to the COVID-19 vaccine to improve acceptance rates. This also provides an opportunity for future studies to comprehensively analyze why behavioral intentions toward COVID-19 vaccines are different from those toward other vaccines.

In several studies, Twitter content analyses have been conducted of health care behaviors other than COVID-19 vaccination. For example, Chew and Eysenbach [14] collected 2009 H1N1-related tweets and identified the resource content posted most often, followed by personal experience, personal opinion, jokes, marketing, and spam. Li et al [33] extracted COVID-19 stigma–related tweets and found ﻿that group labeling, responsibility, and peril tweets disseminated the stigma. Furthermore, several studies have validated the usability of tweets through theory-based content analysis to promote breast cancer promotion programs [16,34]. Our study is the first study to analyze the behavior intention of COVID-19 vaccines through a theory-based content analysis using social media content.

### Limitations

This study has several limitations. First, we only analyzed the behavioral intentions of users on Twitter. Previous studies have shown that health care providers are the primary advocates for vaccination and largely influence vaccination acceptance rates [35-38]. In this platform, we could not distinguish users’ occupations. However, the themes reported in this study could help researchers to develop evidence-based interventions for the general public. To examine the vaccine behavior of health care providers, we will conduct a questionnaire-based survey, and the results from that study could aid the development of clinical guidelines for health workers. This approach of considering the general public separately from health care providers is also recommended by the TIP approach developed by the WHO Regional Office for Europe [24]. Second, there are differences between behavioral intentions and actual vaccine behaviors. However, behavioral intentions have been shown to directly influence actual behaviors [39-41]. Third, Twitter users are considered to be younger and have a higher level of education than the general public [13]. Based on this concern, further qualitative research could be conducted on the older population, those with lower education levels, or those with limited access to the internet.

In future research, a literature review could be conducted to summarize current implementation strategies for COVID-19 vaccine promotion and map them to the themes identified in this study to determine gaps in recent research. The inner mechanism of the adapted COM-B model—the behavior change wheel—could inform evidence-based and theoretical implementation strategies to improve the effectiveness of COVID-19 vaccine promotion programs.

### Conclusion

The study demonstrates the capability of applying the COM-B model to characterize behavioral intentions toward COVID-19 vaccines on the Twitter platform. We successfully generated nine themes of factors that affect behavioral intentions. Positive behavioral intentions were affected by the positive values of vaccination (eg, reduced risk of infection, socioeconomic recovery, return to normal life). In contrast, negative behavioral intentions were associated with attitudes and perceptions about COVID-19 vaccines or the disease itself (eg, underestimation of disease severity, low vaccine effectiveness), values and beliefs (eg, greater belief in the natural immune system), confidence and trust (eg, distrust of government or vaccines), and lack of knowledge. The generated themes could be used to create theory-based and evidence-based implementation strategies to promote COVID-19 vaccines.

## Abbreviations

COM-B: capability, opportunity, motivation–behavior
HBM: health belief model
TIP: Tailoring Immunization Programmes
WHO: World Health Organization
